# Craniocervical junction malformation in a child with Oromandibular-limb hypogenesis-Möbius syndrome

**DOI:** 10.1186/1750-1172-2-2

**Published:** 2007-01-08

**Authors:** Ali Al Kaissi, Franz Grill, Hatem Safi, Maher Ben Ghachem, Farid Ben Chehida, Klaus Klaushofer

**Affiliations:** 1 Ludwig Boltzmann Institute of Osteology at the Hanusch Hospital of WGKK and AUVA Trauma Centre Meidling, 4th Medical Department, Hanusch Hospital, Vienna, Austria; 2 Orthopedic Hospital of Speising, Pediatric Department, Vienna, Austria; 3 Department of Pediatric Orthopedic Surgery-Children Hospital of Tunis, Tunisia; 4 Department of Imaging Studies, Ibn Zohr Institute of Radiology, Tunis, Tunisia

## Abstract

We report a male child with Oromandibular-limb hypogenesis (OMLH), the main features being bilateral sixth and seventh nerve palsies, limb anomalies and hypoplasia of the tongue. Additional features were shortness of the neck associated with torticollis. Radiographs of the cervical spine were non-contributory, but 3D computed tomography (CT) scanning of this area identified: a) congenital hypoplasia of the atlas; b) the simultaneous development of occiput-atlas malformation/developmental defect. To our knowledge, this is the first clinical report assessing the cervico-cranium malformation in a child with OMLH-Möbius syndrome.

## Background

Trauma is the main cause of occiput-atlas abnormality in pediatric acute care practice. Young children are especially vulnerable to this injury because of their small occipital condyles and horizontally oriented atlanto-occipital joints [1]. Trauma can cause injury or rupture of the tectorial membrane and the alar ligaments that allow movement of the cranium relative to the spine [2,3]. We report a 5 years old boy who presented with the full clinical criteria of the Oromandibular-limb hypogenesis (OMLH)-Möbius syndrome. Additional unusual features were short neck and torticollis. Computed tomography (CT) of the craniocervical region was the imaging of choice, and features compatible with occiput-atlas developmental defect and a hypoplastic atlas were identified. Previous reports have discussed the hazardous outcome of occiput-atlas developmental abnormality in the normal pediatric population, but none has related the occiput-atlas injury to a preexisting craniocervical defect. In our knowledge, this is the first report of an association of occiput-atlas developmental abnormality with OMLH-Möbius complex.

## Case report

### Clinical presentation

The patient was born at full term following an uneventful gestation, to a 33 years old mother, with a four-year history of unexplained, primary infertility. At birth, he weighed 2750 g (50 centile), and had a length of 46 cm (3^rd ^centile) and an occipitofrontal circumference (OFC) of 32 cm (50^th ^centile). The mother was married to a 36-year old man who was her first cousin. The family history was non-contributory. No relevant family history of thromophilic disorders or any history of acquired thrombophilia has been identified.

Presentation at birth was vertex, and a number of congenital malformations were noted, including malformations of the hands and feet, bilateral strabismus and a small tongue. Respiratory functions were normal, although syringe feeding was necessary for the first seven months of life.

**Figure 1:**
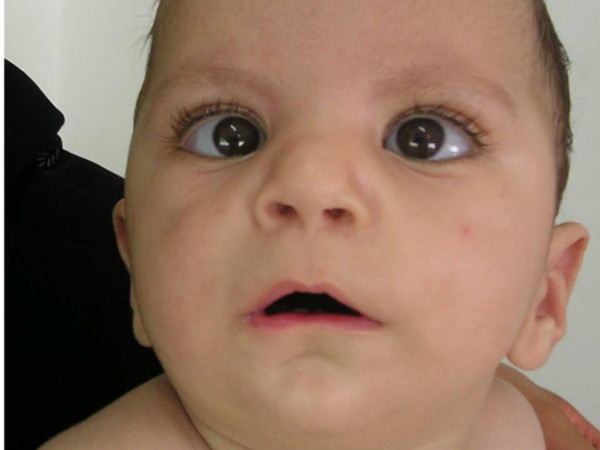
Proband phenotype.

On examination at nine months of age (Figure 1), the child had a normal motor development and hearing, but the coordination was impaired. The body length and weight, and OFC were around the 25^th ^centile. It has been noted an expressionless face (due to bilateral VII nerve palsies), a prominent forehead, depressed nasal bridge, bulbous nose, defective ocular rotation and bilateral strabismus (due to VI nerve palsies). The philtrum was long, with a very thin upper lip and inwardly depressed lower lip, micrognathia, and low set ears. The neck was short with limitations of head movements.

**Figure 2:**
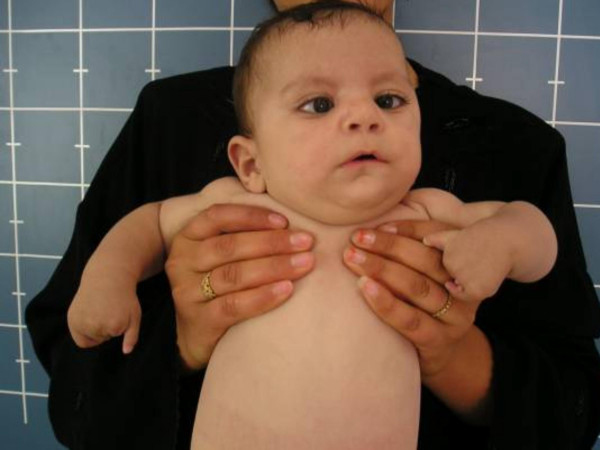
Proband phenotype and hands: Subtotal absence of the phalanges (preservation of the hypoplastic thumbs and hypoplastic 5^th ^fingers, respectively).

**Figure 3:**
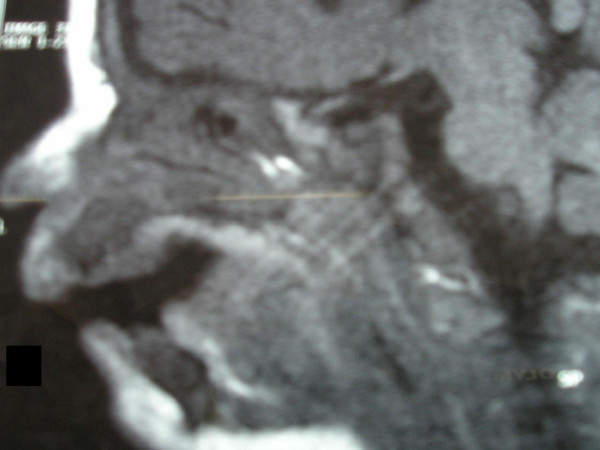
Sagittal MRI imaging, showed markedly hypoplastic tongue.

Although the chest was normal (no associated Poland anomaly), the hands were abnormal with a sub-total absence of the phalanges and preservation of hypoplastic thumbs and hypoplastic 5^th ^fingers (Figure 2). There was also a bilateral adactyly of the feet. The pelvic bones, spinal column and genitalia were normal, as was the ultrasound scan of the kidneys. The sagittal magnetic resonance imaging (MRI) showed a hypoplastic tongue but no associated Arnold-Chiari malformation was detected (Figure 3).

**Figure 4:**
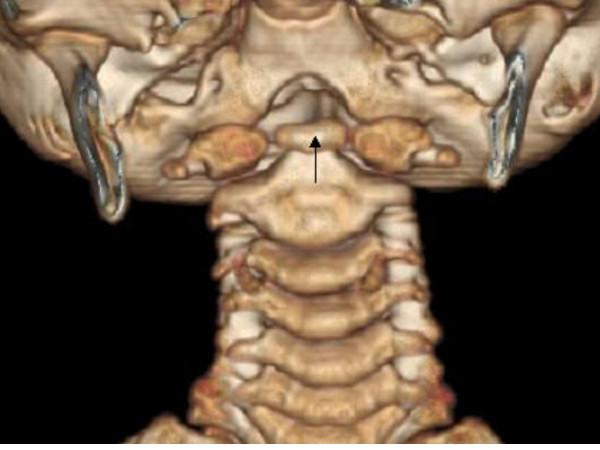
3D reconstruction CT scan ; Hypoplastic anterior arch of the atlas and the impacted os terminale of the odontoid (arrow) between the two halves of the maldeveloped anterior arch of the atlas-the os terminale usually fuses at 12 years of age-this can be confused with fracture.

**Figure 5:**
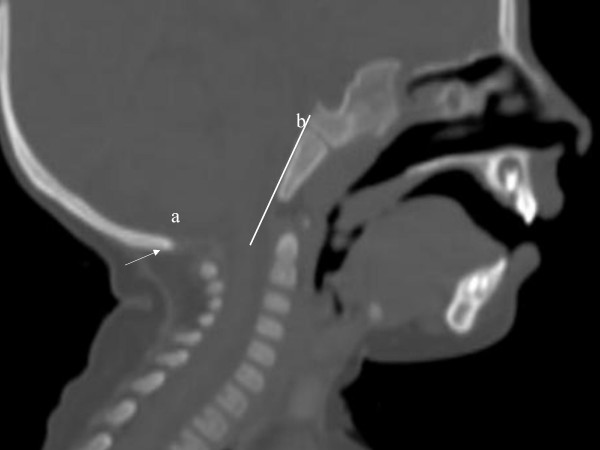
3D sagittal CT scan; Agenesis of the posterior arch of the atlas (arrow-a). Arrow (b) notes the Wachenheim clivus line, which is drawn along the posterior aspect of the clivus toward the odontoid process; in our patient the line does not intersect or is it tangential to the odontoid process. The latter confirms the existence of progressive craniocervical abnormality.

**Figure 6:**
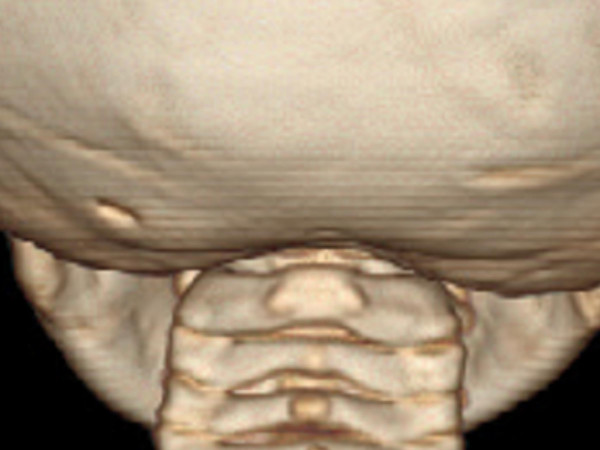
3D reconstruction CT scan showed agenesis of posterior arch of the atlas.

Examination at five years revealed short stature, height being 109 cm (-3SD), and a normal skull circumference of 50 cm. The boy had normal comprehension and receptive language development, but difficulties in expressive language. There were no abnormal neurological findings, except for the cranial nerve palsies (bilateral facial and abducens nerve palsies), which were unchanged. Severe myopia (-8 diopters), shortness of the neck and torticollis have been found and was investigated accordingly (Figures 4, 5, 6). Metabolic screening, chromosomal studies, and hormonal studies of Thyroid-Stimulating Hormone (TSH), Triiodothyronine (T3) and Thyroxine (T4) gave normal results. All basic hematological tests were within normal limits.

### Imaging examination

Cerebral MRI and CT scan showed no associated cerebral abnormalities.

Renal and abdominal ultrasound examination showed normal genito-urinary system.

## Discussion

The hypoglossia/hypodactyly syndrome, the Möbius syndrome, the Hanhart syndrome, the Charlie M syndrome and OMLH are possibly variants of the same condition, and it is often difficult to define the phenotypic boundaries between them [4-11]. There have been a number of studies that reported additional abnormalities but none of these studies have investigated the craniocervical junction [12-16].

Congenital absence or hypoplasia of the posterior arch of the atlas may be associated with several conditions, such as gonadal dysgenesis, and Klippel-Feil, Turner and Down syndromes. In Down syndrome, the hypoplasia of the posterior arch of C1 may lead to a compensatory hypertrophy of the anterior arch of C1 and the spinous processes of C2 [17], whereas in our patient there was a total agenesis of the posterior arch and simultaneous hypoplasia of the anterior arch.

Occiput-atlas abnormality/injury as a result of trauma has been frequently reported in the pediatric emergency practice. It is often fatal, or there may be severe neurological sequelae [1,18-20].

Occiput-atlas injury can also occur in otherwise normal children. Under normal circumstances, there is a minimal rotation between the occiput and atlas, and 50% of cervical rotation occurs between the C1 and C2 articulation [21,22]. However, when a hypoplastic atlas exists, the rotation can be problematic and can lead to abnormal rotation of the upper cervical spine. Rotation then exceeds its normal safe limits, and the spinal cord might be injured. Index that measures the craniocervical integrity is the atlantodens interval (ADI). It is defined as the distance between the anterior aspect of the dens and the posterior aspect of the anterior ring of the atlas, and it should be 5 mm or less [1]. ADI is a marker indicating the normality of the transverse ligament and the alar ligaments, but in patients with hypoplastic atlas, proper measurements cannot be made and the absence of distinctive dens/atlas boundaries make this evaluation most difficult.

We believe that this is the first report of a craniocervical junction malformation in association with OMLH-Möbius syndrome. The hypoplastic atlas abnormality may have an important impact, as it has the propensity to develop into a more profound craniocervical complication. As the conventional radiographic evaluation of the craniocervical junction can be difficult and insufficient to recognize the abnormality (due to rotation and superimposition of the structures), CT imaging is highly recommended.

## Conclusion

This study demonstrates the association of OMLH-Möbius syndrome with torticollis and underlying malformation of the atlas. The latter anomaly may alter the bone-ligament complex and its control on the functions of the craniocervical junction, which is composed of three bones (occipital bone, atlas-axis) and their ligaments. Therefore, the craniocervical junction risks should be carefully assessed and CT is proposed as a valuable neuroimaging technique for craniocervical junction evaluation.

## Abbreviations

OMLH (Oromandibular-Limb Hypogenesis)

OFC (Occipito-Frontal-Circumference)

TSH (Thyroid-Stimulating Hormone)

T3 (Triiodothyronine)

T4 (Thyroxine)

ADI (Atlantodens Interval)

## Competing interests

The author(s) declare that they have no competing interests.

## Authors' contributions

AAK: Own work. Responsible for: a) Writing the manuscript; b) Conception and design; c) Analysis of data.

FBC, HS, and MBG: Data analysis.

FG and KK: Conception and design.
